# Evaluation of Wrist Accelerometer Cut-Points for Classifying Physical Activity Intensity in Youth

**DOI:** 10.3389/fdgth.2022.884307

**Published:** 2022-05-02

**Authors:** Stewart G. Trost, Denise S. K. Brookes, Matthew N. Ahmadi

**Affiliations:** ^1^School of Human Movement and Nutrition Sciences, University of Queensland, Brisbane, QLD, Australia; ^2^School of Exercise and Nutrition Sciences, Queensland University of Technology, Brisbane, QLD, Australia; ^3^Charles Perkins Centre, Faculty of Medicine and Health, School of Health Sciences, The University of Sydney, NSW, Australia

**Keywords:** wearable sensors, children, adolescents, energy expenditure (EE), GGIR, threshold methods, device based monitoring, placement

## Abstract

**Background:**

Wrist worn accelerometers are convenient to wear and provide greater compliance. However, methods to transform the resultant output into predictions of physical activity (PA) intensity have been slow to evolve, with most investigators continuing the practice of applying intensity-based thresholds or cut-points. The current study evaluated the classification accuracy of seven sets of previously published youth-specific cut-points for wrist worn ActiGraph accelerometer data.

**Methods:**

Eighteen children and adolescents [mean age (± SD) 14.6 ± 2.4 years, 10 boys, 8 girls] completed 12 standardized activity trials. During each trial, participants wore an ActiGraph GT3X+ tri-axial accelerometer on the wrist and energy expenditure (Youth METs) was measured directly using the Oxycon Mobile portable calorimetry system. Seven previously published sets of ActiGraph cut-points were evaluated: Crouter regression vertical axis, Crouter regression vector magnitude, Crouter ROC curve vertical axis, Crouter ROC curve vector magnitude, Chandler ROC curve vertical axis, Chandler ROC curve vector magnitude, and Hildebrand ENMO. Classification accuracy was evaluated via weighted Kappa. Confusion matrices were generated to summarize classification accuracy and identify patterns of misclassification.

**Results:**

The cut-points exhibited only moderate agreement with directly measured PA intensity, with Kappa ranging from 0.45 to 0.58. Although the cut-points classified sedentary behavior accurately (> 95%), classification accuracy for the light (3–51%), moderate (12–45%), and vigorous-intensity trials (30–88%) was generally poor. All cut-points underestimated the true intensity of the walking trials, with error rates ranging from 35 to 100%, while the intensity of activity trials requiring significant upper body and/or arm movements was consistently overestimated. The Hildebrand cut-points which serve as the default option in the popular GGIR software package misclassified 30% of the light intensity trials as sedentary and underestimated the intensity of moderate and vigorous intensity trials 75% of the time.

**Conclusion:**

Published ActiGraph cut-points for the wrist, developed specifically for school-aged youth, do not provide acceptable classification accuracy for estimating daily time spent in light, moderate, and vigorous intensity physical activity. The development and deployment of more robust accelerometer data reduction methods such as functional data analysis and machine learning approaches continues to be a research priority.

## Introduction

Accurate assessments of physical activity and sedentary behavior in children and youth are needed to better understand their relationship with health outcomes, as well as evaluate the effectiveness of programs and policies to promote physical activity (1). Historically, researchers conducting field-based studies have relied on self-report methods to assess physical activity (1–3). However, self-report methods are subject to significant social desirability and recall bias (4, 5). Younger children, in particular, have difficulty recalling their past behavior accurately; and struggle to understand the concepts of physical activity frequency, intensity, duration, and type (6). Proxy self-reports completed by parents or caregivers are one solution, but this method is also subject to recall bias since respondents can only report on the time in contact with the child (3, 5). In light of the limitations of self-report methods, device based physical activity measures such as accelerometers have become the preferred method in studies involving children and youth (1, 7, 8). The ActiGraph device is one of the most widely used accelerometer-based motion sensor to quantify physical activity and sedentary behavior in children and adolescents (9, 10).

When first introduced, accelerometers were predominantly worn on the waist or hip in order to capture the body's acceleration and de-acceleration during ambulatory movement. However, over the last decade, the wrist has emerged as a preferable wear location (11). Wrist mounted accelerometers are easier for children to wear for extended periods, thus minimizing missing data due to non-wear (11, 12). Moreover, it enables investigators to evaluate compliance with contemporary 24-h movement guidelines which require concurrent monitoring of sleep duration and quality using wrist actigraphy. Yet, despite the shift to the wrist placement, methods to transform the resultant accelerometer output into predictions of physical activity intensity have been slow to evolve, with most investigators continuing the practice of applying intensity-based thresholds or cut-points (8). With this approach, the relationship between accelerometer output (i.e., proprietary activity counts or gravitational units) is established using linear regression and cut-points delineating established physical activity intensity categories are derived. Another common approach is to use of receiver operating characteristic (ROC) curves to identify cut-points that provide the best possible combination of sensitivity and specificity for differentiating adjacent physical activity intensity categories (13).

To date, at least three investigators have published cut-points for classifying PA intensity from wrist worn ActiGraph accelerometer data in school-aged youth. These cut points and their respective prediction equations are shown in Table 1. Crouter et al. (14) developed cut-points for the dominant wrist using processed count data from the vertical axis (VA) and vector magnitude (VM). Intensity thresholds were determined using both Receiver Operating Characteristic (ROC) curves and linear regression. In similar fashion, Chandler et al. (15) used ROC curve analysis to derive VA and VM cut-points for the non-dominant wrist using accelerometer data collected in children attending summer camp. Finally, Hildebrand et al. (16, 17) derived intensity-based thresholds for unprocessed or “raw” accelerometer signal from the non-dominant wrist based on the Euclidian norm minus one (ENMO) metric. Although the authors reported energy expenditure prediction equations based on linear regression, intensity thresholds were determined using ROC curve analyses.

**Table 1:** Youth specific cut points for the ActiGraph accelerometer worn on the wrist.

**Author**	**Sample**	**Activities**	**Cut-Points**
Crouter et al. (14)	*n* = 18 Range = 8–15 y Mean age = 12.0 y 97 boys, 84 girls	Structure activities: supine rest, watching TV, searching internet, reading, computer games, board games/cards, workout video, vacuuming, sweeping, light cleaning, slow and brisk walking, active video games, playing catch, running, active children's games and sports. Unstructured activity session consisting of watching movies, reading, doing homework, active videogames, soccer, basketball, lifting weights. Cut-points derived using data from the structured activities and cross-validated in data from the unstructured physical activity session.	(CR_ROC_VA) ROC curve analysis Cut points (counts per 5 s): • SED ≤ 105 • LPA > 105 • MPA ≥ 262 • VPA ≥ 565 (CR_ROC_VM) ROC curve analysis Cut points (counts per 5 s): • SED ≤ 275 • LPA > 275 • MPA ≥ 416 • VPA ≥ 778 (CR_REG_VA) METs = 1.592 + [0.0039 (VA counts per 5 s)] Cut points (counts per 5 s): • SED ≤ 35 • LPA > 35 • MPA ≥ 361 • VPA ≥ 1130 (CR_REG_VM) METs = 1.475 + [0.0025 (VM counts per 5 s)] Cut points (counts per 5 s): • SED ≤ 100 • LPA > 100 • MPA ≥ 610 • VPA ≥ 1810
Chandler et al. (15)	*n* = 45 range = 8–12 y Mean age = 9.0 y 22 boys, 23 girls	Resting, enrichment/coloring, walking, playground, splash pad, swimming, shuttle run. LPA defined as > 13.5% of heart rate reserve (HRR). MPA defined as > 50% HRR. VPA defined as > 70% HRR. 10-fold cross-validation	(CH_ROC_VA) ROC curve analysis Cut points (counts per 5 s): • SED <161 • LPA ≥ 162 • MPA ≥ 530 • VPA ≥ 1462 (CH_ROC_VM) ROC curve analysis Cut points (counts per 5 s): • SED <305 • LPA ≥ 306 • MPA ≥ 818 • VPA ≥ 1969
Hildebrand et al. (16, 17)	*n* = 30 Range = 7–11 y Mean age = 8.9 y 16 boys, 14 girls	Lying down, sitting, standing, writing on whiteboard, activity sequence (remove shoes stand, move eight things in a bookshelf, write a sentence, put a paper in an envelope, and sit down), slow treadmill walking, fast treadmill walking, stepping, treadmill running. Leave-one-out cross-validation	(HD_ENMO) ROC curve analysis Cut points (m *g* per 1 s): • SED <35.6 • LPA ≥ 35.6 • MPA ≥ 201.4 • VPA ≥ 707.0

Although the cut-points have been disseminated through the research literature and applied in numerous field-based investigations, no previous study has systematically evaluated their accuracy in an independent sample of youth. Cut-points tend to perform well when evaluated in holdout samples performing the same activities; however, accuracy decreases, often substantially, when tested in independent samples performing different physical activities (9). In addition, cut-points for wrist accelerometer data may provide inaccurate predictions of physical activity intensity because they do not account for the accelerations resulting from upper body and/or arm movements when performing sedentary or non-ambulatory light-intensity physical activities (8, 18).

The absence of an independently conducted validation study simultaneously comparing the performance of wrist cut-points for the widely used ActiGraph accelerometer represents a significant gap in the research literature, given the common use of wrist-worn accelerometers in youth, and the need for standardized approaches to accelerometer data processing. Accordingly, the purpose of this study was to evaluate and compare the classification accuracy of seven previously published sets of youth specific cut-points for wrist worn ActiGraph accelerometer data using energy expenditure, measured via portable calorimetry, as a ground truth measure.

## Methods

### Participants

A total of 18 adolescents (8 girls, 10 boys) participated the study. The descriptive characteristics of the sample were as follows: mean age (± SD) = 14.6 ± 2.4 y, mean body mass index (BMI) percentile = 66.8 ± 25.9%, with 33.3% overweight or obese. Prior to participation, parental written consent and child assent was obtained. The study was approved by the University's Institutional Review Board.

### Study Protocol

Participants completed 12 structured activities over two laboratory visits scheduled within a 2-week time period. The following six activities were completed on visit 1: lying down, handwriting, laundry task, throw and catch, comfortable over-ground walk, and dance. On visit 2, participants completed the remaining six activities: seated computer game, floor sweeping, brisk over-ground walk, basketball, over-ground run/jog, and brisk treadmill walk. Consistent with the recommendations of Welk (19), the selected activities ranged in intensity from sedentary to vigorous, included “lifestyle” physical activities typically performed by children and adolescents, and included both ambulatory and intermittent free-play activities. Each activity trial lasted 5 min except for the lying down trial, which lasted 10 min. A detailed description of the 12 activity trials is provided in Table 2 (9).

**Table 2:** Description of the 12 structured activities.

**Activity type**	**Activity**	**Intensity**	**Description of activity**
Resting	Lying down (LD)	Sedentary	Lie on floor mat or cot in supine position. Awake with arms at side. Instructed to minimize all bodily movements.
Sitting	Handwriting (HW)	Sedentary	While sitting in a chair at a desk, use a ball point pen and a pad of paper to transcribe a standardized written script.
	Computer game (CG)	Sedentary	Seated in a chair at a desk and playing a self-selected online computer game. Game is played by using a keyboard and/or mouse.
Lifestyle /Intermittent	Sweeping floor (SW)	Light/Moderate	Within a 1.5 m x 3 m area, sweep confetti on floor continuously with a broom into a marked 30 x 30 cm box at both ends and repeating.
	Throw and catch (TC)	Light/Moderate	Underarm throw and catch a ball while standing 1.5 to 3.0 m from a research assistant at rate of 15 throws per minute. Distance = 1.5 m for ages 6–7 years, 2.5 m for ages 8–11 years, and 3 m for ages ≥12 years.
	Laundry task (LN)	Light/Moderate	Load a laundry basket with five towels and carry it 3 m; then dump out the towels, fold them, load them back in the basket, carry it back to the original starting spot, and repeat.
	Dance (DA)	Light/Moderate	Follow a simple dance video for children. Routine included simple arm and leg movements.
	Basketball (BB)	Moderate/Vigorous	Shoot a basketball using an 8-ft or regulation hoop. Shoot the ball, rebound, and chase after the ball continuously. Participants instructed to keep the ball within 4.5 m x 4.5 m boundary. Given a new basketball if it leaves the boundary.
Locomotor	Comfortable walk (CW)	Light/Moderate	Walk at a self-selected comfortable speed around the marked perimeter of an indoor gymnasium
	Fast walk (FW)	Moderate/Vigorous	Walk at a self-selected brisk speed around the marked perimeter of an indoor gymnasium
	Brisk walk treadmill (TW)	Moderate/Vigorous	Walk on a treadmill at a speed equal to that achieved during the brisk walking trial.
	Run (RU)	Moderate/Vigorous	Run at a self-selected speed around the marked perimeter of an indoor gymnasium.

### Instrumentation

#### Indirect Calorimetry

Oxygen uptake (VO_2_) during each activity was measured on a breath-by-breath basis using the Oxycon Mobile (Yorba Linda, CA), a lightweight (950 g) portable indirect calorimetry system. A flexible facemask (Hans Rudolph, Kansas City, MO) held in place by a head harness covered the participant's nose and mouth. The mask was attached to a bidirectional rotary flow and measurement sensor (Triple V) to measure the volume of inspired and expired air. A sample tube running from the Triple V to the analyzer unit delivered expired air for the determination of O_2_ and CO_2_ content. Gas exchange responses were interpolated to 1-s intervals and averaged every 15 s using he manufacturer's software. Heart rate was monitored using a Polar H7 heart rate monitoring chest strap. Before each test, the Oxycon unit was calibrated according to manufacturer's guidelines. Flow control and gas calibration were performed using Oxycon's automated calibration system, with the CO_2_ and O_2_ analyzers calibrated against room air as well as to a reference gas of known composition (4% CO_2_ and 16% O_2_). The Oxycon Mobile has been shown to provide valid measures of oxygen uptake over a range of exercise intensities (20).

#### Accelerometry

During each structured activity, participants wore an ActiGraph GT3X+ tri-axial accelerometer (ActiGraph Corporation, Pensacola, FL) on the left and right wrist. The GT3X+ is a small (4.6 x 3.3 x 1.5 cm), lightweight (19 grams) accelerometer-based motion sensor that records time varying accelerations ranging from ± 6 g's. The accelerometer output is sampled by a 12-bit A-D converter at a user specified rate and stored in non-volatile flash memory for subsequent downloading and processing. A sampling rate of 30 Hz was used in the current study. Raw tri-axial acceleration signal was converted to propriety activity counts in the vertical, medio-lateral, and anterior-posterior planes using ActiGraph ActiLife Software (Version 5.8). The vector magnitude (VM) was calculated by taking the square root of the sum of the activity counts squared in each axis. Euclidian norm minus one (ENMO) was derived by calculating the VM of the raw acceleration signal in each axis and subtracting 1 (to correct for the static component of gravity). Negative values were rounded up to zero (21). Prior to calculating ENMO, the raw acceleration signal was calibrated to local gravity using the in-situ autocalibration procedures described by Nadeau et al. (22). The Crouter cut-points were developed for the dominant wrist, while the Chandler and Hildebrand cut-points were developed for the non-dominant wrist. Hence, accelerometer output from the left or right wrist was used depending on the cut-point being evaluated and the child's handedness.

### Data Reduction

Before each test, the ActiGraph and Oxycon units were synchronized to an external timepiece. A customized Visual Basic software program was used to align datetime stamps and calculate mean VO_2_, mean VA counts per 5 second period, mean VM counts per 5 second period, and mean ENMO per 5 second period, recorded between min 2.5 and 4.5 of each structured activity. For the lying down activity, mean VO_2_, mean VA counts per 5 second period, and mean VM counts per 5 second period were calculated from data collected between min 7.0 and 9.0. For each participant, the attainment of steady state was confirmed by inspection of recorded HR and VO_2_. Tolerance levels were ± 5 bpm and 10% for heart rate and VO_2_, respectively. Youth METs, an indicator of absolute intensity, was calculated by dividing mean VO_2_ relative to body mass by resting energy expenditure (REE), where REE was predicted from the participant's sex, age, body mass, and height using Schofield's equation for children aged 3–10 or 10–18 years (23, 24).

### Classification of Physical Activity Intensity

Based on mean VA, VM counts, and ENMO per 5-s period, structured activities were classified as sedentary, light-, moderate-, or vigorous-intensity physical activity using the cut points summarized in Table 1. Structured activities were classified as sedentary, light-, moderate-, or vigorous-intensity physical activity based on absolute intensity as measured by Youth METs. Sedentary activity (SED) was defined as lying or siting posture with a mean energy expenditure <1.5 Youth METs. Light physical activity (LPA) was defined as ≥ 1.5 and <3 Youth METs. Moderate physical activity (MPA) was defined as ≥ 3 and <6 Youth METs. Vigorous physical activity (VPA) was defined as ≥ 6 Youth METs (23).

### Data Analyses

Agreement between measured and predicted physical activity intensity category was evaluated by calculating weighted Kappa statistics. For interpretation of the Kappa coefficients, we followed the ratings suggested by Landis and Koch (25): poor (0–0.2), fair (0.2–0.4), moderate (0.4–0.6), substantial (0.6–0.8), and almost perfect (0.8–1.0). In addition, for each set of cut-points, confusion matrices were generated to summarize classification accuracy within each intensity band and identify patterns of misclassification. Classification accuracy was calculated as the number of correct predictions divided by the number of total predictions. To examine how the mode or activity type impacted classification accuracy, the extent to which each cut-point correctly classified absolute intensity, underestimated absolute intensity, and overestimated absolute intensity was calculated for all 12 structured activities. All data analyses were performed using SAS Version 9.4.

## Results

Of the 216 possible structured activities, complete VO_2_ and accelerometer data were available for 182 trials. Trials were excluded if (1) the accelerometer failed to initialize or download, (2) the Oxycon Mobile malfunctioned, (3) VO_2_ failed to meet the criteria for steady state, or (4) participants were absent, failed to complete the entire trial, or did not follow the instructions. Table 3 displays descriptive statistics for Youth METs, ActiGraph VA counts, ActiGraph VM counts, and ENMO for the 12 structured activities. On average, MET values for lying down, handwriting and computer game fell into the SED category. On average, MET values for throw and catch, laundry, and sweeping fell into the LPA category. On average, MET values for slow walk, dance, brisk walk, and treadmill walking trials fell into the MPA category, whereas the average MET value for basketball and running fell into the VPA category. ActiGraph counts and ENMO during activities requiring significant arm and upper body movement (throw and catch, laundry, and dance) were, on average, higher than those recorded during the walking and running trials, despite having lower energy expenditure.

**Table 3:** Descriptive statistics for Youth METs, vertical axis (VA) counts, vector magnitude (VM) counts, and ENMO for each activity trial.

**Activity**	**Mean METs**	**SD**	**Median VA**	**IQR**	**Median VM**	**IQR**	**Median ENMO**	**IQR**
Lying down	1.3	0.3	0	0–20	0	0–56	21.3	10.7–25.1
Computer game	1.4	0.2	5	0–11	22	5–37	8.5	1.8–21.1
Handwriting	1.5	0.2	7	2–9	35	24–56	0	0–20.7
Laundry	2.4	0.4	1,039	922–1,222	1,583	1,470–1,732	290.1	163.9–334.2
Throw/Catch	2.6	0.7	520	459–624	923	783–1,089	55.9	33.6–83.6
Sweeping	2.9	0.5	394	307–462	573	462–622	26.1	18.9–62.8
Slow walk	3.7	0.6	250	220–287	327	287–381	67.6	30.7–101.2
Dance	4.3	0.8	1,663	1,310–1,903	2,151	1,962–2,418	156.8	130.0–255.5
Fast walk	4.7	0.8	294	253–335	415	364–537	119.5	77.0–181.0
Treadmill walk	5.2	0.9	283	213–337	393	341–521	144.6	96.5–232.0
Basketball	7.2	1.5	1,246	1,045–1,444	1,930	1,692–2,196	450.2	296.5–598.0
Run	9.7	2.3	1,392	1,156–1,477	1,804	1,595–1,983	558.7	396.5–740.5

*Stacked column bar graphs summarizing the extent to which each cut-point overestimated, correctly classified, or underestimated the physical activity intensity category of each structured activity. (LD = lying down, HW = handwriting, CG = computer game, LN = laundry task, TC = throw and catch, SW = sweeping, CW = comfortable walk, DA = dance, FW = fast walk, TW = treadmill walk, BB = basketball, RU = run)*.

**Figure 1:**
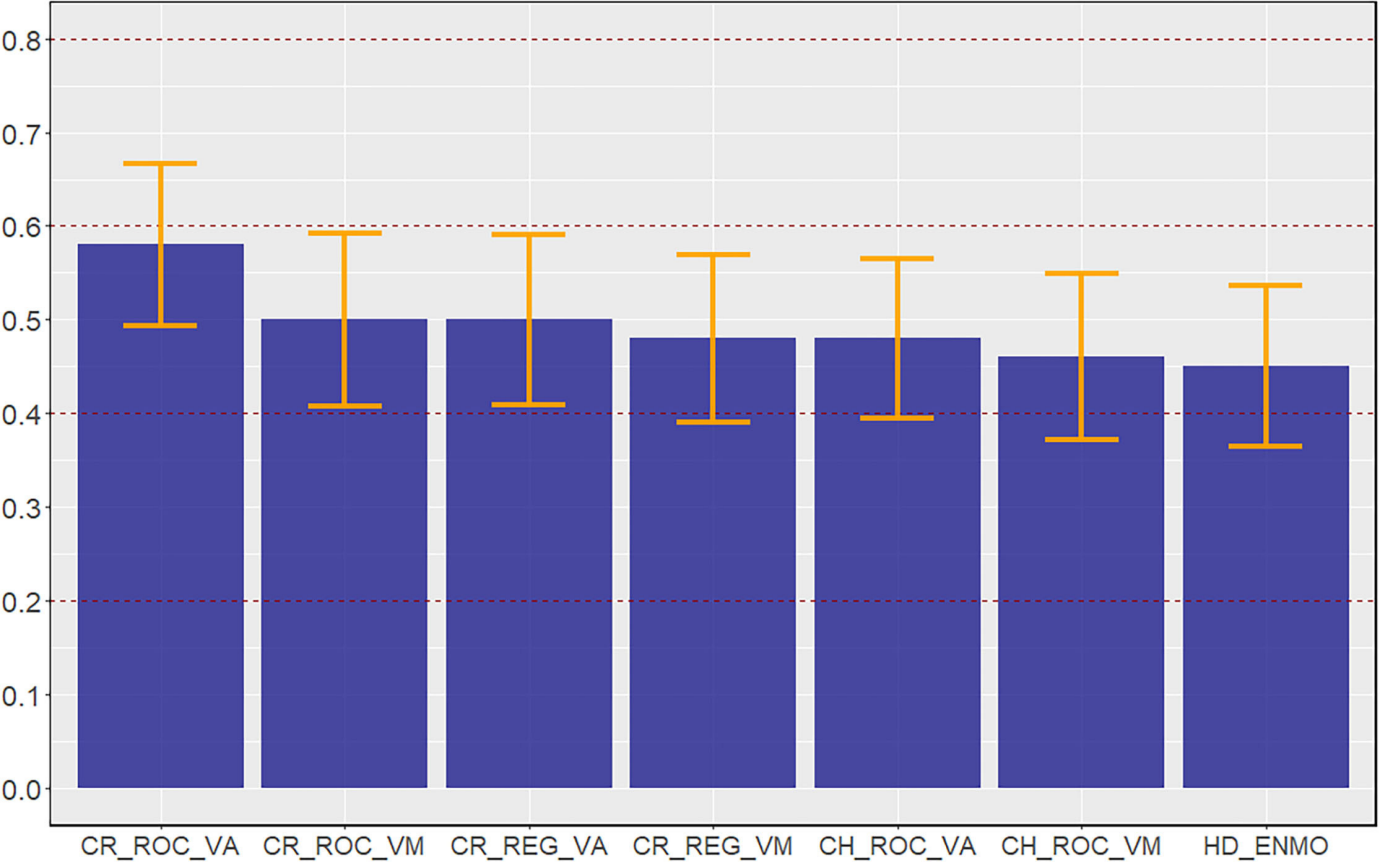
Kappa statistics for the seven youth-specific ActiGraph cut-points for the wrist.

Weighted Kappa statistics and 95% confidence intervals for the seven sets of cut-points are displayed in Figure 1. Applying the rubric of Landis and Koch (25), the cut-points exhibited only moderate agreement, with Kappa statistics ranging from 0.45 (HD_ENMO) to 0.58 (CR_ROC_VA). There were no significant differences in agreement between the cut-points.

**Figure 2:**
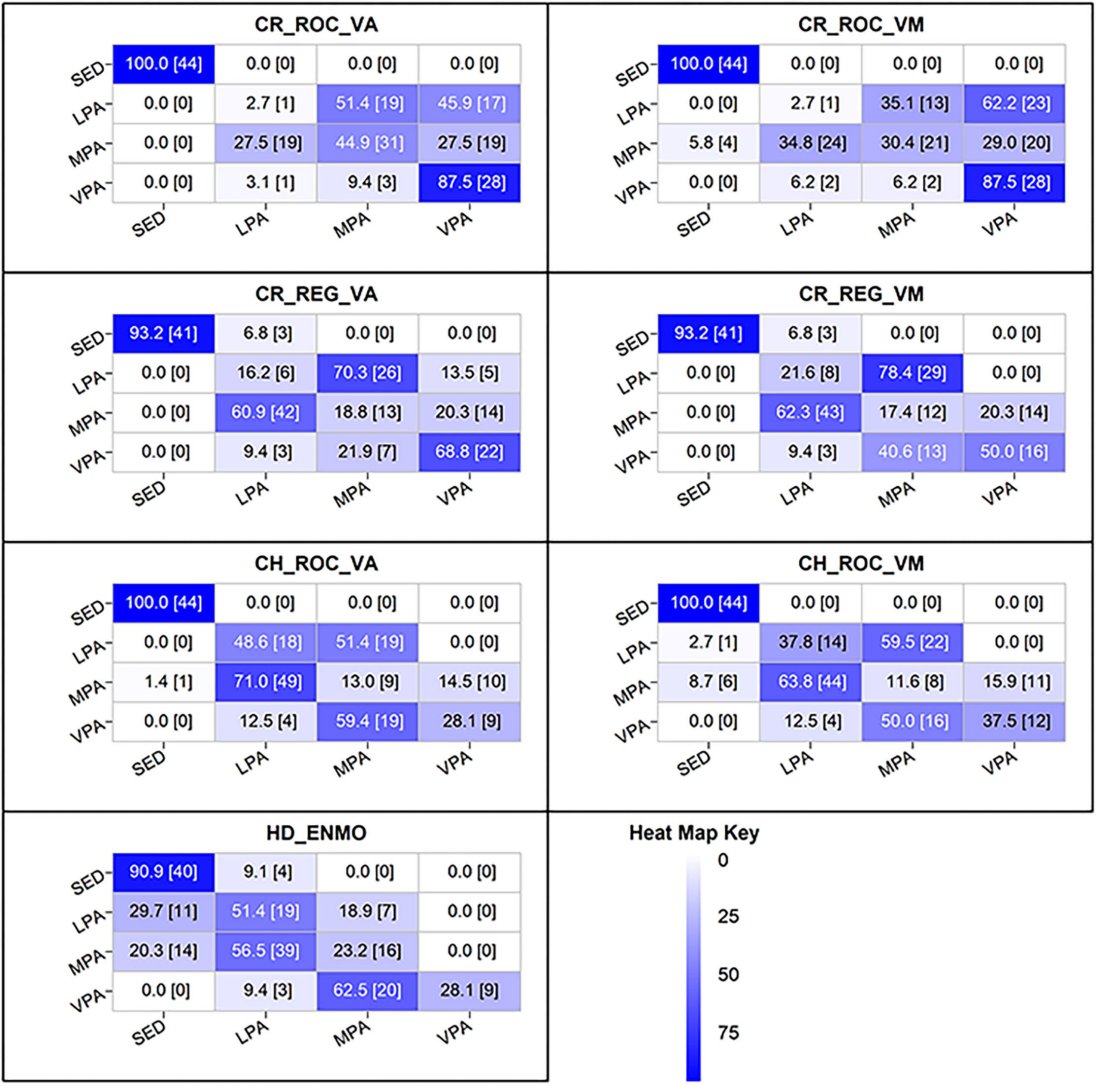
Heat map confusion matrices for the youth specific ActiGraph cut-points for the wrist. Diagonal = correct predictions; Columns = predictions; Rows = measured by portable calorimetry.

Heat map confusion matrices summarizing classification accuracy within each intensity band are displayed in Figure 2. Classification accuracy for the SED trials was consistently high. Four sets of cut-points exhibited 100% accuracy (Crouter ROC VA, Crouter_ROC_VM, Chandler VA, and Chandler VM), with the remaining cut-points exhibiting an accuracy of 90% or greater. When true SED trials were misclassified, they were always misclassified as LPA trials.

Classification accuracy for LPA was extremely poor, ranging from just 3% (Crouter ROC VA/VM) to 51% (Hildebrand ENMO). Among the poorest performing cut-points (accuracy <20%), true LPA trials were misclassified as MPA or VPA. For the remaining cut-points, true LPA trials were consistently misclassified as MPA. Notably, the Hildebrand ENMO cut-point for distinguishing LPA from SED was the only threshold to misclassify a significant proportion of true LPA trials as SED (30%).

Classification accuracy for MPA and VPA was also poor. For the MPA activities, accuracy ranged from just 12% (Chandler VM) to 45% (Crouter ROC VA). True MPA activities were misclassified as LPA or VPA, with the majority misclassified as LPA. Notably, the Hildebrand EMNO cut-points misclassified 20% of the true MPA activities as SED. The intensity of the VPA activities was consistently underestimated as MPA or LPA. With the exception of the Crouter ROC VA and VM cut-points (88% accuracy), between 30 and 70% of true VPA activities were misclassified as MPA or LPA.

**Figure 3:**
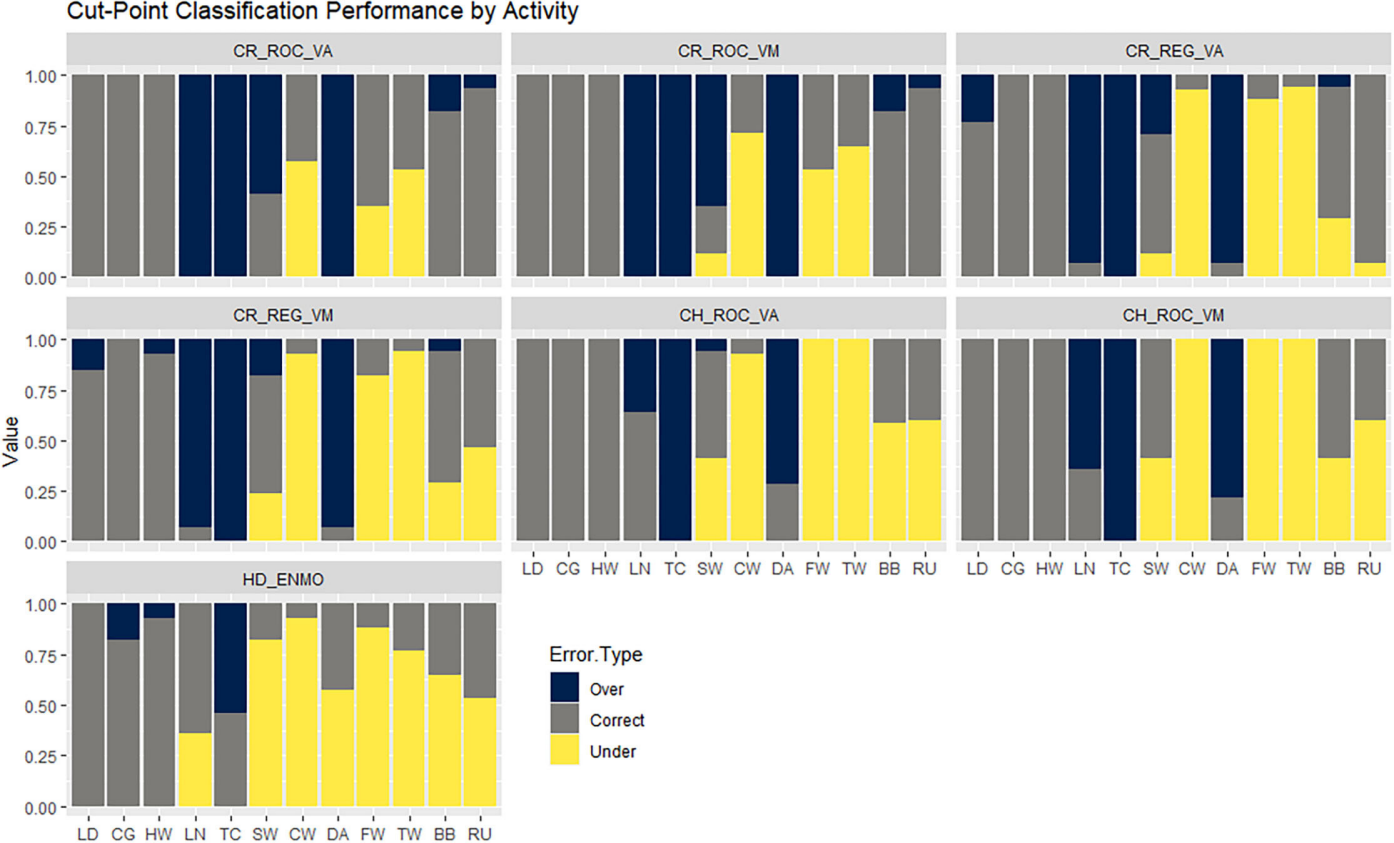
Stacked column bar graphs summarizing the extent to which each cut-point overestimated, correctly classified, or underestimated the physical activity intensity category of each structured activity. (LD = lying down, HW = handwriting, CG = computer game, LN = laundry task, TC = throw and catch, SW = sweeping, CW = comfortable walk, DA = dance, FW = fast walk, TW = treadmill walk, BB = basketball, RU = run).

Figure 3 summarizes the extent to which the cut-points overestimated, correctly classified, or underestimated the intensity of each structured activity. All seven sets of cut-points underestimated the true intensity of walking, with error rates ranging from 57 to 100% for slow walking, 35 to 100% for brisk walking, and 53 to 100% for brisk walking on a treadmill. In contrast, the cut-points consistently overestimated the true intensity of non-ambulatory activities requiring significant upper body and/or arm movements (throw and catch, laundry task, and aerobic dance). Notably, for this activity type, the overestimation rate for the Crouter cut-points exceeded 90%. For activities at the high end of the intensity spectrum (basketball, running), the cut-points consistently underestimated physical activity intensity. The exception to this pattern was the Crouter ROC cut-points, which overestimated the intensity of a small percentage (7%) of the basketball and running activities. As noted above, all seven sets of cut-points correctly classified the intensity of sedentary activities (lying down, handwriting, and videogame).

## Discussion

The current study evaluated the classification accuracy of previously published youth-specific cut-points for wrist-mounted ActiGraph accelerometers. The results clearly demonstrate that wrist cut-points perform poorly when applied to new accelerometer data from an independent sample. Cut-points for the wrist exhibited only moderate agreement with directly measured physical activity intensity, and with the exception of true sedentary activities, physical activity intensity was misclassified 40–60% of the time. Of concern, light-intensity activities involving extensive upper body and/or arm movements were consistently misclassified as MVPA, while the intensity of moderate-to-vigorous intensity ambulatory activities such as walking and running was consistently underestimated. These findings indicate that wrist cut-point methods for the ActiGraph accelerometer data are not valid for quantifying time in light and moderate-to-vigorous intensity physical activity and that other accelerometer data processing strategies should be applied.

The poor performance of the wrist cut points was not unexpected considering that, for many human activities, acceleration recorded at the wrist is not a reliable indicator of physical activity intensity. Threshold approaches assume that the magnitude of acceleration, expressed in proprietary activity counts or gravitational units, is strongly correlated with the rate of energy expenditure (18, 26). This assumption may be reasonable when the accelerometer is positioned close to the body's center of mass on the participant's waist or hip. However, when the accelerometer is worn on the wrist, this assumption is no longer tenable. The energy cost of non-ambulatory activities with significant arm or wrist movement will be overestimated, while the intensity of moderate-to-vigorous activities with limited or constrained arm movement will be underestimated (27, 28). Such misclassification errors were clearly visible in the current study. All seven sets of cut-points overestimated the intensity of activity trials involving significant arm or wrist movement (throw and catch, laundry, dance), while everyday moderate intensity activities such as brisk walking were routinely misclassified as light-intensity physical activity. The intensity of vigorous activities such as running and playing basketball was consistently underestimated and misclassified as moderate or light intensity activity. Notably, activity counts and ENMO recorded during the laundry trial were, on average, three times higher than those recorded during the brisk walking trial, despite requiring only half the energy expenditure. Median activity counts recorded during the dance activity trial were 20–30% higher than those recorded during the vigorous intensity running and basketball trials (7–10 METs), despite having an average energy cost of just over 4 METs. Collectively, these findings underscore the need for alternative modeling approaches for wrist accelerometer data.

For more than a decade, research leaders in device-based measurement have been calling for a shift away from cut-point methods and the use of machine learning accelerometer data processing approaches (29, 30). This call has led to the development of several youth-specific machine learning physical activity classification and energy expenditure regression models for wrist accelerometer data and other wear locations (31–38). When evaluated in independent samples of children, machine learning models for wrist accelerometer data produce more accurate predictions of physical activity intensity than cut-point methods (36, 37). Yet, despite such findings, the uptake of machine learning methods among public health researchers and sports scientists has been minimal, primarily because their implementation requires basic to intermediate programming skills and specialized software. Indeed, most authors now provide links to the final prediction model, along with snippets of code and sample datasets (28, 31, 32), making it is possible for end users without specialist coding skills to implement machine learning models in open-source platforms such as R and Python. Nevertheless, it is acknowledged that in the absence of open-source software tools (e.g., graphic user interfaces) to simplify the application of machine learning models, the uptake of machine learning methods will be limited, and researchers will continue to use flawed cut-point methods. Considering the significant misclassification errors observed in the current study, the development of user-friendly software tools for implementing machine learning models represents a critical research priority.

Because the Hildebrand cut-points serve as the default option for estimating physical activity intensity in the popular GGIR accelerometer data processing package in R (39), the performance of these cut-points warrant closer inspection. Within our sample, the Hildebrand cut-points misclassified 30% of the light intensity trials as sedentary behavior and underestimated the intensity of moderate and vigorous intensity trials over 75% of the time. Of concern, 20% of the moderate intensity activity trials, including those that involved walking, were misclassified as sedentary behavior, while the true intensity of the brisk walking, basketball, and running trials was consistently underestimated. While GGIR end users can specify their own intensity-related thresholds and use thresholds established for other gravitational unit types, our results suggests that time in intensity estimates based on the default Hildebrand ENMO cut-points should be interpreted with extreme caution GGIR users should consider using other metrics and/or data reduction approaches available in GGIR (40) or apply validated youth-specific machine learning models for wrist accelerometer data.

The current study has several strengths. First, to account for individual differences in the energy cost of performing a given physical activity, energy expenditure, measured by portable indirect calorimetry was used as a criterion measure of physical activity intensity. This was an important design feature because previous wrist accelerometer validation and calibration studies involving children have mostly relied on one-size-fits-all intensity ratings based on direct observation (28, 41, 42) or predictions of intensity from concurrently worn hip-mounted accelerometers (43, 44). Second, classification accuracy was examined in children completing a variety of activities that ranged in posture, tempo, absolute intensity, and amount of arm movement. This contrasts with previously published studies that evaluated wrist cut-points using predominantly sedentary and light-intensity activities with little or no arm movements (27, 45).

Offsetting these strengths were several limitations. First, due to the burdensome nature of the data collection protocol, our sample size was relatively small. However, our dataset was more than adequate to address the aims of the study. The 182 structured activities available for analysis provided a 95% confidence interval width of 0.17, which was within the 0.20 difference between adjacent categories of agreement proposed by Landis and Koch (25). Nevertheless, our findings require replication in larger, more diverse samples of children and adolescents. Second, because the lying down activity trial did not follow established protocols for measuring resting energy expenditure, predicted resting energy expenditure was used to calculate Youth METs. However, our reported MET values were in close agreement with previously published values (23). Third, to maximize internal validity and obtain steady-state measures of energy expenditure, participants completed a series of controlled structured activities which may not fully replicate the movement behaviors undertaken by free-living children and adolescents. Therefore, additional research is needed to evaluate the classification accuracy of ActiGraph wrist cut-points for school-aged children under true free-living conditions. It is worth noting that two recent studies involving free-living preschool-aged children found that cut-points for the wrist and hip were associated with significant misclassification error (32, 42). Therefore, it is unlikely that wrist cut-points for school-aged children will perform differently under true free-living conditions.

In conclusion, previously published ActiGraph cut-points for the wrist, developed specifically for school-aged youth, do not provide acceptable classification accuracy for estimating daily time spent in light, moderate, and vigorous intensity physical activity. Public health researchers and sports scientists using wrist mounted accelerometers to quantify movement behaviors in school-aged youth are urged to adopt alternative accelerometer data processing methods such as functional data analysis (40, 46) or machine learning approaches based on time and frequency domain features in raw acceleration signal (35).

## Data Availability Statement

The data supporting the conclusions of this article will be made available by the authors, without undue reservation.

## Ethics Statement

The studies involving human participants were reviewed and approved by Oregon State University Institutional Review Board. Written informed consent to participate in this study was provided by the participants' legal guardian/next of kin.

## Author Contributions

ST conceived the design of the study, data collections procedures, data analysis, and manuscript writing. DB contributed to conceptualization of the paper and manuscript writing. MA contributed to the conceptualization of the paper, provided input into the data analysis, and contributed to manuscript writing. All authors contributed to the article and approved the submitted version.

## Funding

The research was supported by a grant from the US National Institutes of Health (5R01HD055400-03). The funder played no role in the execution of this research study.

## Conflict of Interest

The authors declare that the research was conducted in the absence of any commercial or financial relationships that could be construed as a potential conflict of interest.

## Publisher's Note

All claims expressed in this article are solely those of the authors and do not necessarily represent those of their affiliated organizations, or those of the publisher, the editors and the reviewers. Any product that may be evaluated in this article, or claim that may be made by its manufacturer, is not guaranteed or endorsed by the publisher.
